# Hidden variable models reveal the effects of infection from changes in host survival

**DOI:** 10.1371/journal.pcbi.1010910

**Published:** 2023-02-22

**Authors:** Jake M. Ferguson, Andrea González-González, Johnathan A. Kaiser, Sara M. Winzer, Justin M. Anast, Ben Ridenhour, Tanya A. Miura, Christine E. Parent

**Affiliations:** 1 Department of Biology, University of Hawaiʻi at Mānoa, Honolulu, Hawaii, United States of America; 2 Institute for Modeling Collaboration and Innovation, University of Idaho, Moscow, Idaho, United States of America; 3 Department of Biology, University of Florida, Gainesville, Florida, United States of America; 4 Department of Biological Sciences, University of Idaho, Moscow, Idaho, United States of America; 5 Department of Mathematics, University of Idaho, Moscow, Idaho, United States of America; 6 Institute for Interdisciplinary Data Sciences, University of Idaho, Moscow, Idaho, United States of America; Washington State University, UNITED STATES

## Abstract

The impacts of disease on host vital rates can be demonstrated using longitudinal studies, but these studies can be expensive and logistically challenging. We examined the utility of hidden variable models to infer the individual effects of infectious disease from population-level measurements of survival when longitudinal studies are not possible. Our approach seeks to explain temporal deviations in population-level survival after introducing a disease causative agent when disease prevalence cannot be directly measured by coupling survival and epidemiological models. We tested this approach using an experimental host system (*Drosophila melanogaster*) with multiple distinct pathogens to validate the ability of the hidden variable model to infer per-capita disease rates. We then applied the approach to a disease outbreak in harbor seals (*Phoca vituline*) that had data on observed strandings but no epidemiological data. We found that our hidden variable modeling approach could successfully detect the per-capita effects of disease from monitored survival rates in both the experimental and wild populations. Our approach may prove useful for detecting epidemics from public health data in regions where standard surveillance techniques are not available and in the study of epidemics in wildlife populations, where longitudinal studies can be especially difficult to implement.

## Author summary

Effectively monitoring disease outbreaks requires determining the proportion of diseased individuals and the consequences of disease through time. The ecological fallacy is the statistical assumption that groups are homogenous when they are not. The fallacy can arise in epidemiology when studying populations composed of both diseased and healthy individuals. The average mortality in the population will not accurately describe either a diseased or a healthy individual. This is a challenge for monitoring disease outbreaks since many forms of monitoring data are collected at the population level. Here, we apply a modeling approach that accounts for disease transmission at the individual-level when data are collected at the population-level. The approach works by comparing the pattern of mortality through time in a population with a disease to a fully healthy one. The observed rate of change in mortality through time informs the model of the infection process. We validated our approach using an experimental system (*Drosophila melanogaster* infected with different viral pathogens). We then applied the method to study an outbreak of phocine distemper in harbor seals. Our results show that the individual-level effects of disease can be determined from population-level data.

## Introduction

Longitudinal studies that track individuals through time are the benchmark for detecting the effects of disease on survival and reproduction because they can directly link a change in an individual’s infection status to a change in their vital rates. Despite their power, longitudinal studies tend to be relatively rare, especially those concerning wildlife, due to the prohibitive costs and logistics of tracking individuals. Alternatives to longitudinal studies, such as tracking groups instead of individuals, can illustrate differences among populations; however, these comparisons cannot explicitly determine the individual-level impacts of the disease. The inability for inferences at the group level to be valid at the individual level is commonly known as the ecological fallacy.

Hidden variable models, also called latent-variable models, explicitly link processes that are not directly observed to the measured random variables, providing an effective pathway to model complex biological processes using aggregate data [1]. Commonly used hidden variable modeling approaches in biology include latent variable regression [2], hidden Markov [3], state-space models [4], and structural equation models [5]. In epidemiology, state-space approaches have been successfully used to link the observed number of deaths due to disease to the underlying disease dynamics [6] and distinguish seasonal and epidemic dynamics in public-health surveillance data [7,8]. Coupling hidden variable models to inexpensive and commonly collected forms of data, such as population-level measurements of vital rates, could offer a powerful new approach for retroactively determining the individual-level effects of disease on populations.

Monitoring vital rates, such as survival, is a common approach for managing wildlife populations. These measurements have been used to provide insights into the past causes of change in population growth [9] and to forecast the effects of potential future environmental conditions on population growth [10]. In the context of disease studies, coupling vital rates to disease prevalence has been used to determine how bovine tuberculosis, a mild infection at the individual-level, can lead to large reductions in the population growth of African buffalo [11]. More recent work has extended this approach to use vital rate and prevalence measurements to identify the impacts of interactions between multiple diseases [12]. Thus, while monitoring vital rates is a potential resource for detecting the effects of disease, the current usefulness of these data for answering epidemiological questions is contingent on having detailed information on individuals infection status.

Here, we determined whether the effect of infection on a host’s survival rates could be inferred without direct measurements of infection prevalence. Our approach coupled the observed survival rates to a model that predicted the infected status of individuals, the demographic rates of both infected and uninfected individuals and the probability of becoming infected. Conceptually, we are able to infer the survival rates of infected and uninfected individuals by comparing the temporal change between the observed pre- and post-infection survival rates.

We first demonstrate proof-of-concept of our modeling approach on a tractable experimental host-virus system of *Drosophila melanogaster* and the associated Drosophila C virus (DCV) and Drosophila X virus (DXV) where longitudinal surveys were impossible to perform due to the destructive nature of specimen sampling. We then apply the modeling technique to a phocine distemper outbreak in the Netherlands where weekly mortality data are collected but individuals are not tested for disease or virus presence. In both scenarios, disease incidence could not be directly measured but systematic changes in population-level vital rates were observed post-infection.

## Materials and methods

### Survival experiment

#### Fly line and husbandry

We used the Oregon R wild-type strain of *Drosophila melanogaster* from Carolina Biology Supply. The stock and experimental flies were kept in an incubator on a 12-hour day/night cycle at 25°C. We housed the stock flies in 25 x 95 mm polystyrene vials (Genesee Scientific) with 10 ml of Jazz-Mix Drosophila Food referred here as fly food (Fisher Scientific) prepared following the manufacturer’s instructions. As described in [13], we bleached freshly laid eggs to clear any potential chronic infections before performing the viral challenges.

#### Infecting pathogens

We used Drosophila C (DCV) and Drosophila X (DXV) viruses as infecting pathogens. DCV is a +ssRNA virus that belongs to the Dicistroviridae family. It has been found in natural populations of at least eight different *Drosophila* species [14,15] and has been extensively used as a model system to study the antiviral response in *D*. *melanogaster* [16–19] as well as the ecology and evolution of viral infections in *Drosophila* [20–22]. While it is possible for oral inoculation by DCV to result in systemic infection, it is more often associated with a local immune response and results in low mortality rates compared to intra-thoracic injections [17,23]. DXV is a dsRNA virus from the Birnaviridae family and is considered to be a cell culture contaminant [24]. It is genetically similar to the dsRNA Eridge virus isolated from natural populations of fruit flies in France and the UK [15]. Although the effects and pathology of DXV are not as well understood as DCV in *Drosophila*, it has been shown that infections by microinjection induce anoxia sensitivity, exhibited by increased rates of mortality when exposed to CO_2_ [24–26].

We used the Charolles strain of DCV isolated from a laboratory population in 1972 [27] and obtained from Dr. Marta L Wayne (University of Florida). We obtained the DXV stock from Dr. Louisa Wu (University of Maryland). Both DCV and DXV were cultured in Schneider’s Drosophila Line 2 (S2 cells) and titrated to a tissue culture 50% infectious dose of 2x10^9^ TCID_50_/ml as previously described [28]. Viral stocks were kept in 250 μl aliquots at -80°C.

#### Oral infection and survival monitoring

We infected flies through an oral inoculation protocol meant to resemble a natural route of infection [29–31]. We collected newly emerged flies in vials containing clean fly food and left them to age for three days in an incubator on a 12-hour day/night cycle at 25°C to reach maturity. We next starved the mature flies for four hours by placing them in empty polystyrene vials and then transferred them into new vials supplemented with a 1.9 cm diameter circular piece of Whatman filter paper (Cat No 1001 150) placed in the bottom. The filter paper was loaded with 100 μl of viral or negative control mixture. We prepared the viral mixture for infections by combining 50 μl sucrose 25%, 225 μl DCV or DXV virus stock, 225 μl Schneider’s Drosophila Medium (Genesee Scientific), and 10 μl of edible red dye. For the negative control (i.e., mock) trial, 450 μl of Schneider’s Drosophila Medium was used in addition to the 50 μl sucrose 25% plus 10 μl of edible red dye. After six hours of letting the flies feed on one of the mixtures, only the flies showing a red belly (indicating recent ingestion of viral or mock mixture) were transferred into vials containing clean fly food.

**Fig 1:**
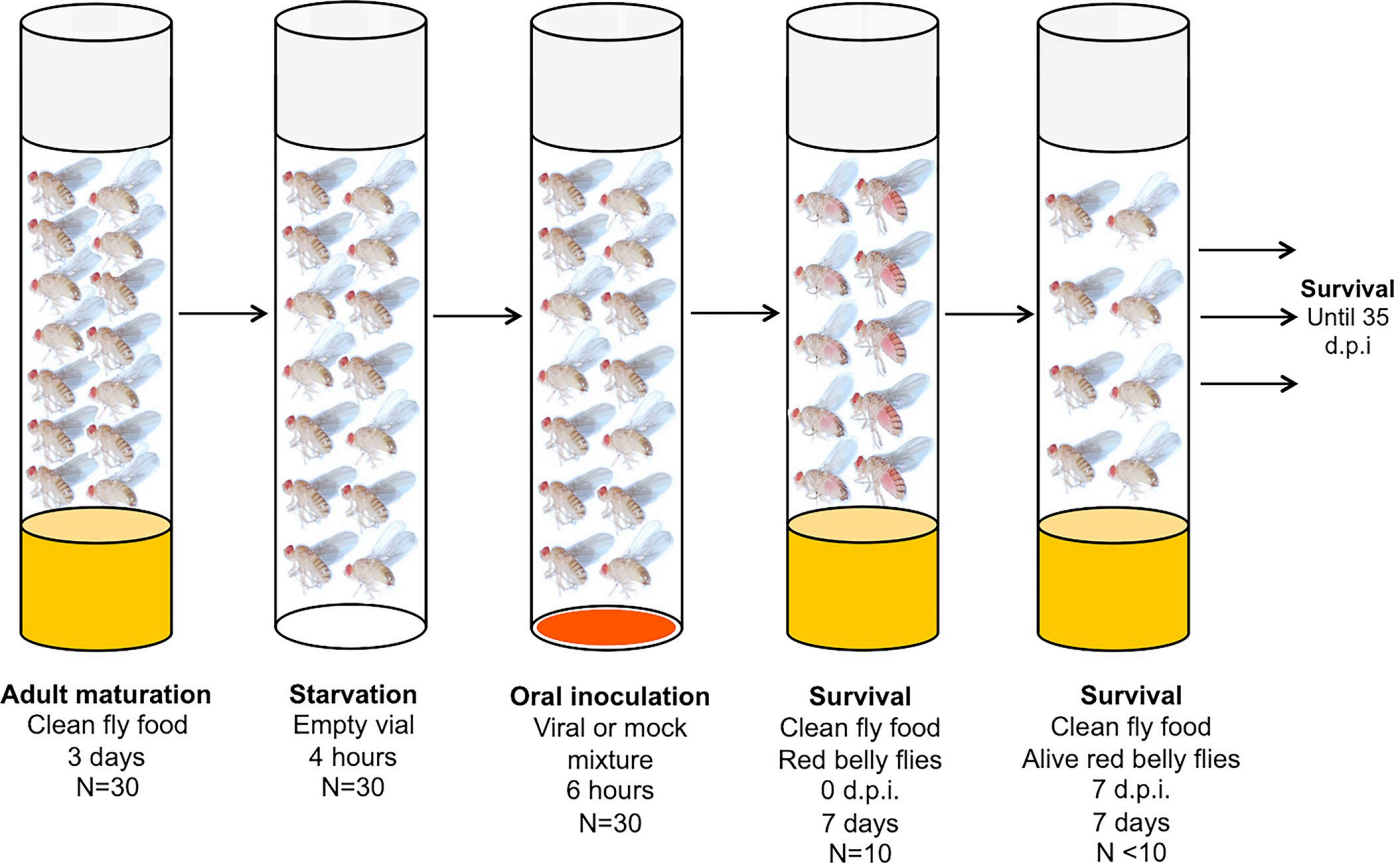
Diagram of the oral DCV or DXV infection protocol in *Drosophila melanogaster*. First, 15 males and 15 females were placed in a clean vial containing fly food for 3 days to reach complete maturation. Flies were next transferred into an empty vial to starve for 4 hours. Flies were then placed into new vials for 6 hours, where flies fed from either a virus or mock mixture that contained red dye. After this inoculation period, 10 flies (5 males and 5 females) showing a red belly, were transferred into a new vial containing food (without any pathogen). Finally, the number of dead and alive flies was scored on a daily basis over a period of 35 days and alive flies were transferred into clean vials containing fly food every 7 days.

Each infection treatment (DCV or DXV) and negative control (Mock) consisted of 10 replicate vials, each vial housing 5 red belly females and 5 red belly males for a total of 100 flies per treatment or negative control. We placed the experimental vials in the incubator and counted the number of dead flies in each tube daily over 35 days. Alive flies were transferred into new vials containing clean food every seven days to avoid the emergence of offspring. Deceased flies were not collected immediately after a mortality event to avoid potential stress from repeated anesthetization of the experimental populations and we were not able to collect deceased flies from the 7-days old vial because the corpses were either decomposed or buried deep in the fly food. We illustrate these experimental steps in Fig 1.

#### Fly survival models

**Fig 2:**
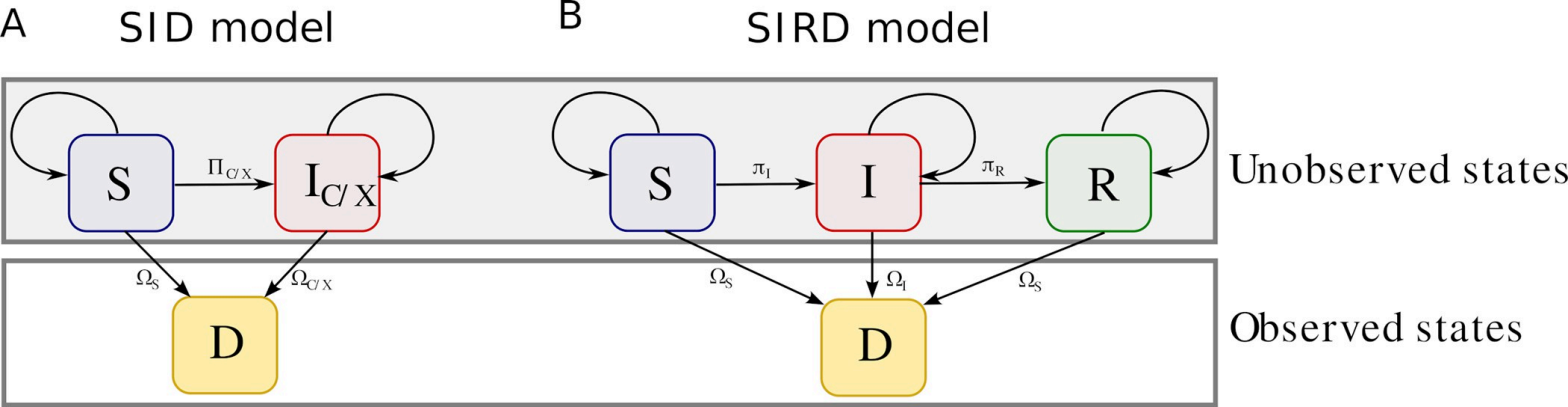
State transitions for the SID model (panel A) and the SIRD model (panel B). Blue boxes denote the susceptible state (denoted as *S*). Red boxes in the SID model denote the infected state including either DCV (*I*_*C*_) or DXV (*I*_*X*_). The red box in the SIR model is the state for individuals infected with phocine distemper (*I*), while the green box is the recovered state (*R*). The probability of death is denoted as *Ω*(*t*), with appropriate subscript, and the probability of transitions from one state to another are denoted with Greek letters. Lines that begin and end in the same state denote the proportion of individuals that stay in the same state the following time step. Thus, in the SID model the probability of remaining in state S from time *t*−1 to time *t* is 1−*Ω*_S_(*t*)−Π_*C*/*X*_(*t*). Since D is an absorbing state, all individuals remain in this state.

We analyzed the effects of DCV and DXV on the mortality rates of flies by treating the experimental data as mixtures of individuals with three different possible states (i.e., susceptible, infected, or dead). We started our experimental populations with all flies ingesting the viral mixture, however, since determining the state of infection in these flies required destructive sampling we could not determine which of these exposed flies became diseased. However, experimental evidence suggests that individual flies orally infected with DCV start clearing the virus as soon as two days post-infection [17,23]. Because we did not observe the dynamics typical of the recovery phase, such as an increase in average survival near the end of the experiment, we assumed that our experiment data are composed of a mixture of an unknown number susceptible and infected individuals. To resolve this unknown mixture, we used an epidemiological model to predict the numbers of susceptible and infected flies throughout the 35-day experiment. Flies selected for the experimental treatments were known to be directly exposed to DCV or DXV through the consumed medium. Subsequent infections likely follow a fecal- oral route of transmission [14,16]. Thus, we modeled the disease and population dynamics of the flies using a susceptible-infected with death (hereafter, SID) model. We modeled transmission with two components. The first was constant daily infection probabilities arising from environmental transmission (parameter denoted as *π*_0,*C*/*X*_). The second component was a density-dependent component that depends on the numbers of susceptible and infected each day (parameter denoted as *π*_*C*/*X*_). Together, these terms combined to give the total probability of infection of a susceptible individual as $\Pi_{C/X}(t)=\pi_{0,C/X}+\pi_{C/X}I_{C/X}(t)$. The population size in each vial also changed through time through death, accounted for in our model by age-dependent survival probabilities. All transitions used in infection experiments are illustrated in Fig 2A. Our model assumed that all individuals in a vial began in the susceptible class (*S*(*t* = 0) = 10) with no individuals in any infected class (*I*_*C*/*X*_(*t* = 0) = 0). Susceptible individuals could become infected with DCV or DXV with the daily probability of infection (*π*_*C*/*X*_), or they could die with the time-dependent daily probability of death (*Ω*(*t*)).

The daily probability of death, *Ω*(*t*), was the probability of dying between day *t*−1 and day *t*, given that the individual survived up to day *t*−1. We denote the probability of an individual in state *Z* dying on day *t* in vial *j* as *Ω*_*Z*,*j*_(*t*), modeled as $logit\;\left(\Omega_{Z,j}(t)\right)=\alpha_{Z}+\beta_{age}\cdot t+\beta_{transfer}\cdot Transfer+\psi_{T,j}$. The time-independent mortality term, *α*_*Z*_, was assumed to be state-dependent denoted as *α*_*S*_ for susceptibles, *α*_*C*_ for DCV-infecteds, and *α*_*X*_ for DXV-infecteds. The regression parameters *β*_*age*_ and *β*_*transfer*_ were assumed to be independent of infection status; *β*_*age*_ accounted for the age of the fly and *t* denotes the days since the beginning of the experiment and *β*_*transfer*_ is the effect of the number of days after the last transfer (*Transfer* is the number of days, from 0 to 6, since the last vial transfer), as described in the experimental methods. These transfers occurred each week and we observed increases in fly mortality with the amount of time between transfers. This mortality pattern likely was due to the medium becoming sticky over time and trapping flies. Finally, *ψ*_*T*,*j*_, was a treatment- and vial-specific random-effect, assumed to follow a normal distribution with unknown variance, where the treatment was either the mock, DCV-exposed, or DXV-exposed flies. In addition, we fitted a model with no random effect and compared the model fits using the deviance information criterion (DIC) [32].

The total number of infected individuals, *I*_*C*/*X*_(*t*), where the subscript *C* denotes DCV and *X* denotes DXV, on day *t* was modeled as the number of susceptibles on the previous day that survived and became infected plus the existing infecteds from the previous day that survived. The total number of deaths on day *t*, *D*(*t*), was then the sum of the deaths from both susceptible and infected individuals. Thus, the expected numbers of susceptibles, infecteds, and deaths on day *t* in a population exposed to a virus given the previous day’s population are:

$$S_{j}(t)={(1-\Omega }_{S,j}(t))\cdot S_{j}(t-1)–\Pi_{C/X,j}(1-\Omega_{S,j}(t))\cdot \dot{S_{j}(t-1)}$$

$$I_{C/X,j}(t)=({1-\Omega }_{C/X,j}(t))\cdot I_{C/X,j}(t-1)+{\Pi_{C/X,j}(1-\Omega }_{S,j}(t))\cdot S_{j}(t-1)$$

$$D_{j}(t)=\Omega_{S,j}(t)\cdot S_{j}(t-1)+\Omega_{C/X,j}(t)\cdot I_{C/X,j}(t-1).$$

We modeled the number of flies that survived daily in the mock experiment as a binomial distribution with probability of death following *Ω*_*S*,*j*_(*t*) and size parameter *N*_*j*_(*t*−1), the observed number of individuals alive at the end of the previous day in vial *j*. For the infection model, the observed probability of death depended on both the probabilities of death of infected and susceptible individuals, weighted by their relative proportions. Thus, the probability of death in vial *j* followed a binomial distribution with probability *D*_*j*_(*t*)/(*S*_*j*_(*t*−1)+*I*_*C*/*X*,*j*_(*t*−1)) and size parameter *N*_*j*_(*t*−1). Specifications for all priors in this model are given in S1 Table.

In order to determine whether our SID model was a reasonable model of disease dynamics, we tested it against a model with no SID dynamics. This treatment-specific model assumed that all individuals in an experiment had the same chance of dying each day, thus, unlike the SID model there was no differentiation between infected and noninfected individuals in the vial. In this treatment-specific model the daily mortality probability was modeled following the survival models for *Ω*_*Z*,*j*_(*t*) described above, including the effects of day, days since the last vial transfer, and a vial-specific random effect; however, this model did not include any differences between susceptible and diseased individuals. Thus, all individuals in a vial were assumed to have the same daily mortality probability. We also compared our SID model with both environmental and density dependent transmission to a model with just environmental transmission (i.e., *π*_*C*/*X*_ = 0), since *a priori* we believed this to be the dominant mode of transmission. We compared the fits of these models using the deviance information criterion (DIC).

We fit all fly survival models in JAGS [33] using an adaptive phase of 10^3^ draws, a burn-in of 10^4^ draws, followed by 10^6^ draws from the posterior, thinning these draws by every 100 for a total of 10^4^ draws from the posterior distribution. We examined the posteriors graphically for convergence and checked the Gelman and Rubin convergence diagnostic [34]. We reported the posterior mean and the standard deviation of the posteriors. In order to determine whether flies in the experimental treatments differed from the mock flies, we performed pairwise comparisons between posterior distributions of the time-independent mortality terms (*α*_*S*_, *α*_*C*_, and *α*_*X*_). In order to assess parameter identifiability, we generated 10,000 datasets using the mean posterior estimates from the SID model. We then re-estimated the model for each of these simulated datasets and recorded the mean parameter estimate for each parameter.

### Harbor seal study

#### Stranding surveys

Phocine distemper virus is closely related to the canine distemper virus in the Paramyxoviridae family; both pathogens are in the same genus as measles virus [35]. An outbreak of phocine distemper in 2002 was detected in the North Sea population of harbor seals. First detected on Anhholt Island in Denmark in May, the virus spread into the Netherlands with the first detection on June 16, presumably by the highly mobile gray seals who share haul-outs with harbor seals [36]. The 2002 outbreak cost an estimated 30,000 harbor seals their lives, making this the largest recorded mass mortality event ever in marine mammals [36].

Data on seal stranding events were obtained from [37] and [38], who used a public database, waarneming.nl, that contained weekly stranding data collected by seal rescue centers in the region. Stranding events may or may not be lethal, though rescued individuals are removed from the population and brought to local seal rescue centers. We used WebPlotDigitizer software [39] to acquire data from published figures from [37] and [38], rounding all digitized values to the nearest integer value. The Dutch outbreak ended by December the same year as indicated by the return of stranding rates to pre-epidemic levels [37], which resulted in 25 weeks of stranding data. We also obtained annual harbor seal strandings from [38] for the three years before the 2002 outbreak (1999–2001) and the three years following the outbreak (2003–2005) to determine the average weekly stranding rates in the absence of the phocine distemper outbreak. We limited our study to these years because the harbor seal population was relatively constant over that period of time [38].

#### Phocine distemper model

As in the fly experiments, we could not directly observe disease incidence in seals, only the change in strandings due to disease. It is the change in rate of strandings from the background rate that provides information on epidemic parameters. Because we were only able to observe strandings, deaths that did not lead to a stranding were not incorporated into this analysis. We model all stranding events as deaths, since seals were removed from the wild population.

We modeled seal disease dynamics using a susceptible-infected-recovered with death (SIRD) model (Fig 2B) that linked the latent infectious state of individuals in the population to changes in stranding rates through time. This model also incorporated the possibility of recovery from disease. In contrast to the fly survival model described above, which was parameterized using probabilities, we parameterized the SIRD model in terms of time scales to facilitate comparison with previously published results.

The probability of infection of a susceptible individual, *π*_*I*_, was modeled using a discrete frequency-dependent model [40], $\pi_{I}(t)=1–exp\left(-\frac{I(t-1)}{\beta N(t-1)}\right),$ where *β* determines the transmission period, determining how long it takes for susceptibles to become infected each week. The probability of recovery was modeled as $\pi_{R}=1–exp\left(-\frac{1}{\gamma }\right)$, where *γ* determines the time to recovery of an infected individual, while the probability of stranding of an infected (*I*) individual was $\Omega_{I}=1–exp\left(-\frac{1}{\omega }\right)$, where *ω* determines the time it takes for an infected individual to become stranded. We calculated the expected time for these transition events to occur by assuming a geometric distribution for the transition event with a probability of success given by estimated transition probability (either *π*_*R*_ or Ω_*I*_). We assumed the population size at the beginning of the outbreak was *N*(0) = 5400 individuals, following estimates developed from aerial surveys [38]. We treated the initial number of infecteds in the population, *I*_0_, as an estimated parameter since it has been hypothesized that the disease may have been introduced by gray seals from Denmark and subsequently passed to harbor seals throughout the North Sea [36].

We estimated the stranding probability of susceptible and recovered individuals (denoted as *Ω*_*S*_) using the annual number of strandings from the three years prior to the 2002 outbreak (1999–2001) and the three years following the outbreak (2003–2005). Because population size estimates were not available each year, we assumed that the population size was always 5400. We further assumed that stranding rates were constant through the year so that the estimated weekly number of strandings was the annual count divided by 52. The probability of stranding was then the average weekly number of strandings divided by 5400.

Combining these rates as in Fig 2B, our SIRD model structure was given by:

$$S(t)={(1-\Omega }_{S})\cdot S(t-1)–{\pi_{I}(t)\cdot (1-\Omega }_{S})\cdot \dot{S(t-1)}$$

$$I(t)=({1-\Omega }_{I})\cdot I(t-1)+{\pi_{I}(t)\cdot (1-\Omega }_{S})\cdot S(t-1)-{\pi_{R}\cdot (1-\Omega }_{I}(t))\cdot I(t-1)$$

$$R(t)={(1-\Omega }_{S})\cdot R(t-1)+{\pi_{R}\cdot (1-\Omega }_{I})\cdot I(t-1)$$

$$D(t)=\Omega_{S}\cdot S(t-1)+\Omega_{S}\cdot R(t-1)+\Omega_{I}\cdot I(t-1)$$

with initial conditions given by *S*(0) = 5400−*I*_0_, *I*(0) = *I*_0_, and *R*(0) = 0. We assumed that the number of strandings on day *t* followed a negative binomial distribution with mean *D*(*t*) and size parameter *τ*. Specifications for all priors in this model are given in S2 Table. We used the same specifications and posterior checks as used in the fly SID models. For all parameter estimates, we report the posterior mean and the standard deviation. In order to assess parameter identifiability, we simulated 10,000 datasets from SIRD model using the posterior mean estimates. We then re-estimated the model for each of these simulated datasets and recorded the mean parameter estimates of each parameter.

## Dryad DOI

https://doi.org/10.5061/dryad.jm63xsjf9 [56].

## Results

### Fly survival

**Fig 3:**
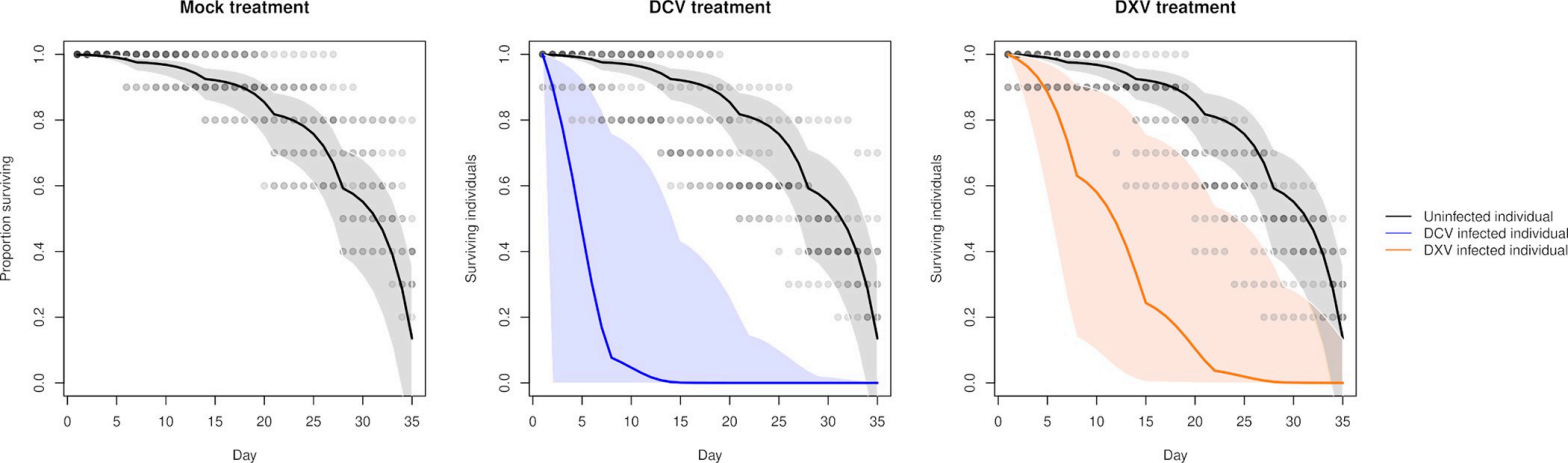
Points indicate the proportion of surviving individuals in each vial. Transparency is used to indicate overlapping values. Colored lines are the mean estimated survival probabilities that is composed entirely of uninfected flies (panel A), infected with DCV (panel B), or infected with DXV (panel C). Shaded regions denote the 95% credible interval of the mean.

We detected similar mortality rates by the end of the survival experiment (day 35 post-infection) in all treatments. Total mortality was 60% in the mock treatment, 64% in the DCV treatment, and 70% in the DXV treatment. Observed treatment-level mortality rates throughout the experiment are presented in Fig 3 and indicate that the temporal pattern in mortality differed between treatments.

In our comparison of a model with SID dynamics to the treatment-specific model, we found strong evidence that the SID model fit the data better than the model with treatment-specific (i.e., no SID dynamics) mortality rates (ΔDIC = 12.4). We also found the SID model with both environmental and density dependent transmission fit the data better than a model with environmental transmission only (ΔDIC = 3.3). Comparing SID models with and without random effects had nearly identical fits (ΔDIC = 0.9), indicating very little variation among vials within the same experimental treatment. Our inspection of the point and interval estimates of survival and infection probability were nearly identical between these models. Here, we present the results for the full model with random effects but these results hold for the model without random effects as well.

**Fig 4:**
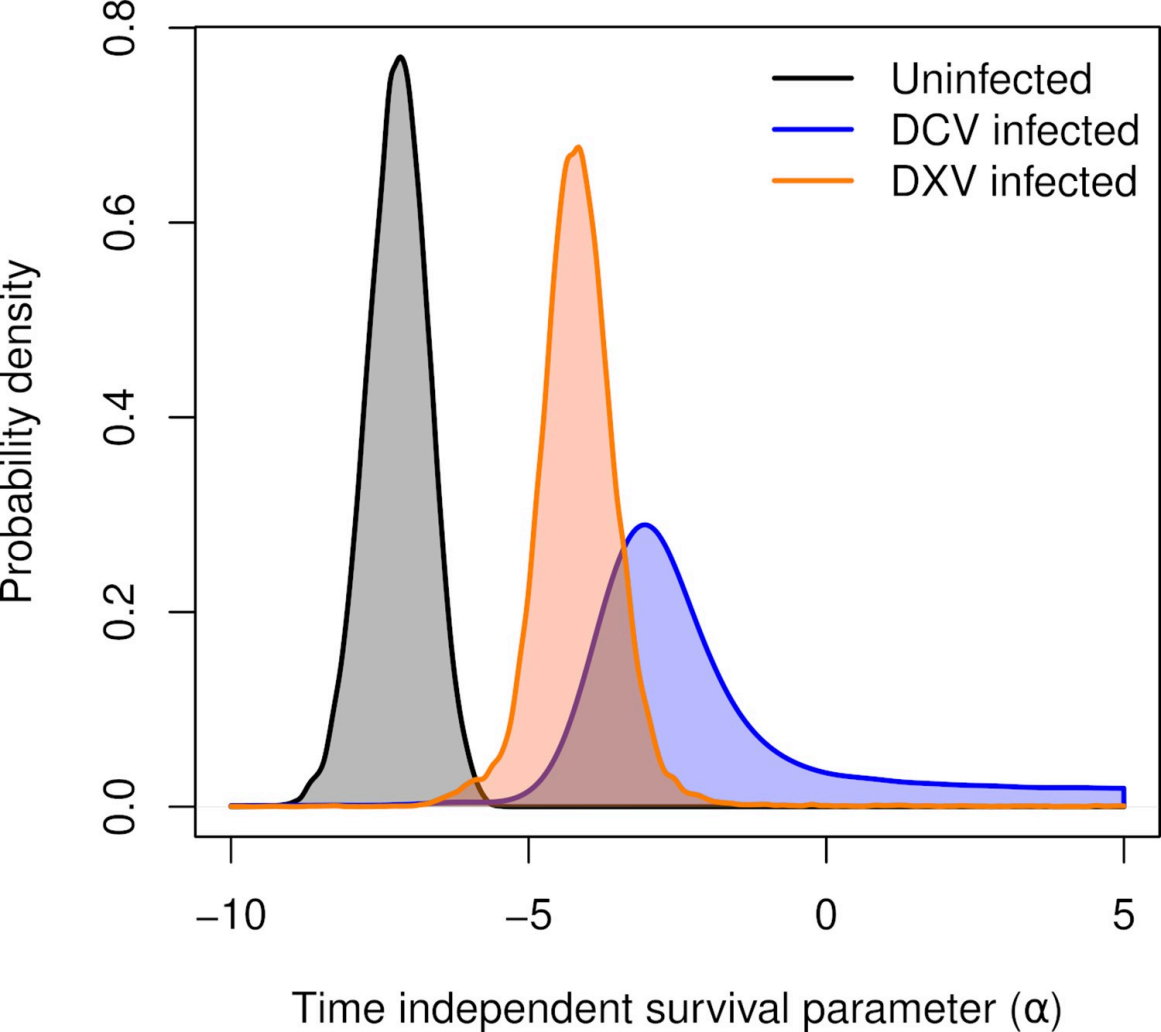
Posterior distributions for the time-independent survival of uninfected individuals (*α*_*S*_), DCV infected individuals (*α*_*C*_), and DXV infected individuals (*α*_*X*_).

We found that the time-independent of the logit daily survival parameters (reported as mean (standard deviation)) for an uninfected individual (${\hat{a}}_{S}=-7.1\;(0.5)$) differed from DCV (${\hat{a}}_{C}=-2.3\;(6.1)$) or DXV (${\hat{a}}_{X}=-4.0\;(1.7)$) infected individuals (Fig 4). These estimates corresponded to odds ratios of death for the infected versus the susceptible classes was $\frac{{\hat{\Omega }}_{C}}{{\hat{\Omega }}_{S}}=127.4\;(308.7)$ for DCV and $\frac{{\hat{\Omega }}_{X}}{{\hat{\Omega }}_{S}}=21.5\;(89.9)$ for DXV. In both cases the credible interval of the odds ratio did not cover one, indicating a significantly higher odds of death for flies that are infected by DCV or DXV. The estimated time-dependent probability of mortalities are illustrated in Fig 3. In pairwise comparisons between the posteriors of the time-independent component of survival, we found that the 95% high density intervals (hereafter, HDI) of the difference between infected and uninfected estimates did not overlap zero while in the comparison between the infected classes the 95% HDI’s did overlap zero (Fig 4). Thus, while we could statistically distinguish between a susceptible individual and an individual infected with DCV or DXV, we could not distinguish between the effects of DCV and DXV infections.

Our estimates also indicated that survival declined as flies age (${\hat{\beta }}_{age}=0.11\;(0.02)$ per day) and the time between transfers increased (${\hat{\beta }}_{transfer}=0.28\;(0.02)$ per day). Additionally, our estimates of the probability of infection in the SID model found that the daily probability of becoming infected with DCV (environmental transmission probability, ${\hat{\pi }}_{0,C}=0.007\;(0.007),$ density dependent transmission probability, ${\hat{\pi }}_{C}=0.03\;(0.02)$) or DXV (environmental transmission probability, ${\hat{\pi }}_{X}=0.005\;(0.007)$, density dependent transmission probability, ${\hat{\pi }}_{X}=0.04\;(0.02)$) were statistically similar, as the 95% HDI overlapped 0). All other parameter estimates are reported in S1 Table, while the simulation results (S1 Fig) indicated low bias in all model estimates.

### Phocine distemper outbreak

**Fig 5:**
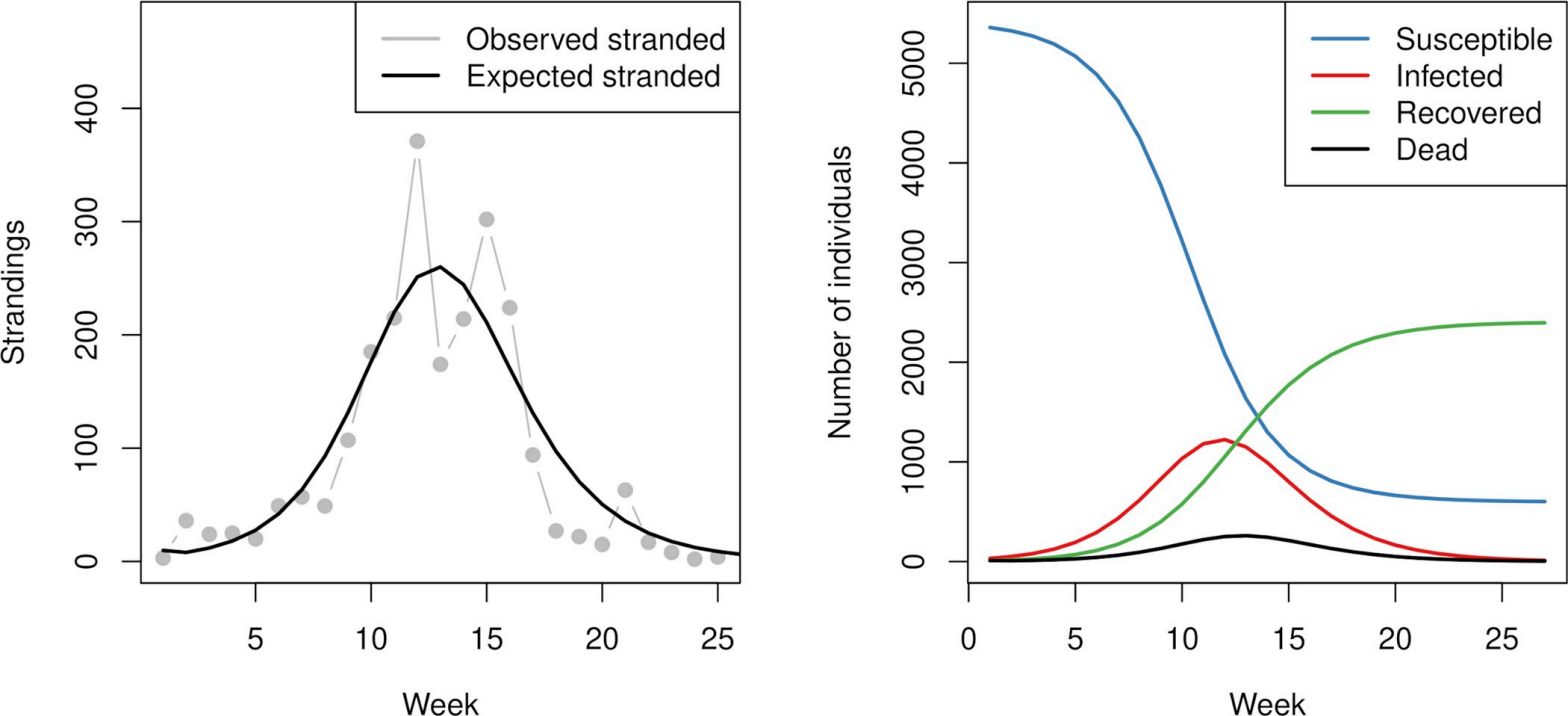
Panel A illustrates the observed (grey points) and expected number of strandings from the SIRD model (black line). All deaths are assumed to lead to strandings. Panel B illustrates the expected number of susceptible (blue), infected (red), recovered (green), and dead (stranded) seals (black) over the course of the outbreak.

After validating our approach in the fly system, we applied the same protocol to the SIRD model. Our estimated weekly stranding probability of an uninfected individual (reported as mean (standard deviation)) was ${\hat{\Omega }}_{S}=2.2x10^{-4}\;(0.4x10^{-4})$, while the weekly probability that an infected individual became stranded was estimated as ${\hat{\Omega }}_{I}=0.19\;(0.17)$, giving an odds ratio of $\frac{{\hat{\Omega }}_{I}}{{\hat{\Omega }}_{S}}=1244\;(745)$ and the credible interval of this ratio did not cover 1. Thus, infected seals were nearly one-thousand times more likely to get stranded than uninfected seals. Our model adequately explained the location of the peak of infection, estimated to occur in week 13, and the subsequent decline of the number of strandings in the harbor seal population (Fig 5A). Over the 25 weeks of observations, our model simulated that over 80% of the population became infected with phocine distemper at some point, for those infected there was a 54% chance of becoming stranded (Fig 5B). This is consistent with mortality estimates of 60% in the 1988 outbreak of phocine distemper in harbor seals in the North Sea [41,42]. Finally, we estimated that the number of strandings at the end of the epidemic was 2198 individuals, about 40% of the total population.

We estimated that the mean time to recovery was between 2.6 as 3.1 weeks (with $\hat{\gamma }=3.1\;(1.7)$ reported as mean (standard deviation)), consistent with the mean length of the infected period of 15 days reported in [42]. The mean time to death once infected was estimated as 3.6.2 weeks ($\hat{\omega }=3.4.2\;(2.4)$ weeks), similar to previous reports of 3 weeks until death in dogs infected with canine distemper as reported in [43], though this has not been directly measured in pinnipeds. We estimated the transmission period as $\hat{\beta }=0.9\;(0.1)$ weeks and the initial number of infecteds as $\hat{I_{0}}=50.0\;(20.0)$ individuals. The posterior distribution of the period of transmission, $\hat{\beta }$, was strongly correlated both with the estimated time until death, $\hat{\omega }$, (*r* = 0.80) and estimated the initial number of infecteds, ${\hat{I}}_{0}$ (*r* = 0.82). Posterior estimates of all parameters are reported in S1 Table. Results from simulating datasets from the SIRD model using the estimated parameters indicated that parameter bias was low for all parameters other than the mean time to recovery. Here we found a bias in this parameter of 0.5. Applying this bias-correction yielded an estimate of $\hat{\gamma }=2.58\;(1.7).$

## Discussion

Our results show that hidden variable modeling approaches can be useful for estimating the per-capita effects of disease outbreaks from commonly collected forms of monitoring data such as population abundances. Differences in survival in our experiment were subtle but strong enough to jointly estimate per-capita vital rates and disease prevalence. The increasing discrepancy between experimental treatments and the mock treatment over the course of the experiment allowed the SID model to determine how disease prevalence changes over time due to transmission of the pathogen. This in turn allowed us to accurately determine the per-capita effects of disease. However, in this case we could only estimate the proportion of individuals that became sick enough to affect their mortality because we did not observe a recovery phase of the outbreak that would provide information on the recovery rates of infected individuals despite the relatively short time to clear the virus that has been reported previously [17,30]. We may have not observed a recovery phase in our data because the length of the experiment may not have been sufficient to observe the decline in the number of infecteds that occurs after peak infection. This decline depends on not just the recovery rate but also the rate of infection. Alternatively, if infected individuals developed chronic health issues that affected their survival post-recovery, we would not expect to observe a recovery phase.

In contrast to the fly experiment where recovery was not observed, we were able to reconstruct both the 2002 phocine distemper epidemic and the population’s subsequent recovery. This recovery phase allowed us to determine the rate of recovery providing additional information about how many individuals became infected but did not die. Thus, while we were able to estimate the contribution of nonlethal infections in the phocine distemper outbreak we were unable to detect their effects in the DCV and DXV experiments. In both examples, we found epidemiological parameter estimates consistent with past studies. Thus, this modeling approach can yield accurate per-capita estimates of the effects of disease on vital rates such as survival from observational data. We also note that while we focused on survival in this study, it is also possible to model other monitored vital rates such as fecundity, to determine impacts of disease.

Another approach developed to determine the influence of disease on survival is the method of excess mortality [44]. This uses individual data to model the survival of patients relative to a background population. The method has found extensive use for determining the impact of cancer [45,46], infectious disease [47], impacts of surgery [48], and even natural disasters [49] on an individual’s survival. In contrast with our approach, the method of excess mortality requires that each individual’s infection status at the time of death is known while in the latent variable approach this status is unknown.

Our models were able to successfully infer the individual-level effects of infection from population-level survival data; however, two conditions must be met for hidden variable models to successfully estimate the impacts of disease on vital rates. First, the overall effect of infection on the population’s vital rate must be strong enough to detect a statistical difference from an uninfected population. Second, the underlying epidemiological dynamics must generate systematic variation in the degree of prevalence linked to the per-capita change in prevalence predicted by the epidemiological models. When both conditions are met, the individual-level effects of disease can be determined. In cases where these conditions are not met or when additional precision is needed, additional information on disease prevalence can be used to infer individual-level effects of disease. For example, tools such as serology, histopathology, immunohistochemistry and molecular diagnostic techniques such as qPCR and RT-qPCR have been commonly used to confirm the presence of phocine distemper virus in infected marine mammals [50,51]. These data, if properly collected, could be used to independently estimate disease prevalence. A limitation of the approach is that we found the estimated parameters tended to have significant uncertainty. One way to improve estimates would be to use past studies to develop informative priors to constrain estimates of epidemiological parameters [52].

While hidden-variable models show significant promise, there are also several important limitations when using this indirect approach. One important tradeoff is that inferences made using these models may be quite sensitive to the underlying modeling assumptions. Another potential factor to consider when applying these models is knowledge about disease introduction. We knew both the time of disease introduction and the number of initial infecteds in the fly experiment. In the harbor seal example, we assumed knowledge of the time of introduction based on previous reports but not the initial number of infecteds. Estimating the initial number of infecteds is approximately equivalent to estimating the time of the disease introduction when the number of initial infected is known. However, if both pieces of information are unknown, it is unclear whether the parameters could be uniquely estimated.

The nature of the disease needs to be considered when developing models to estimate different epidemiological parameters and to properly contextualize the results of these models. For example, the route of infection can modulate the severity of the infection in fruit flies. Intrathoracic injection elicits a systemic infection that results in the death of all flies by day 9 post-infection. In contrast, oral infections have found mortality rates of less than 10% by day 15 post-infection [23]. In line with previous studies [17,30], our DCV survival results suggest low mortality rates estimated by day 15 post-infection. Like DCV, intrathoracic injection with DXV results in 100% mortality by day 10 post-infection [26]. However, we found similar patterns of low mortality rates when the flies are orally infected with DXV. To our knowledge, our study is the first to determine the effects of oral inoculation with DXV on fly survival. These differences between the mortality rates elicited by route of infection are likely to be due to the type of interaction established between pathogen and host and to the immune response mounted by the host. For example, it has been demonstrated that the amount of DCV virus ingested by the flies is significantly higher than the viral dose injected to them, however, orally infected flies are able to start clearing DCV without using the classic antiviral mechanisms such as RNAi used by the fly immune system when infected by injection. Interestingly, orally infected flies start clearing the infection despite clear evidence of viral replication [23].

We expect that our hidden variable modeling approach will be valuable for wildlife health surveillance. While several methods exist to detect wildlife epidemics early, many rely on explicitly surveying infection levels [53,54] and may require substantial resources to implement. Our modeling approach could detect emerging epidemics from existing vital rate data that are often collected for management purposes. Proposed monitoring databases, such as the Marine Mammal Health Monitoring and Analysis Platform [55] have emerged with the goal of collecting monitoring data of mortality events at daily to weekly time intervals. Coupling latent variable models to these data will allow us to potentially explain anomalous mortality events due to disease and forecast the effects of disease outbreaks in real-time, providing powerful tools for interventions in sensitive populations.

## Supporting information

S1 Table Parameter definitions and estimates for the fly SID models. (DOCX)

S2 Table Parameter definitions and estimates for the seal SIRD models. (DOCX)

S1 Fig Histograms of 10,000 estimated values under a simulation experiment which generated data using from the mean of posterior estimates of the SID model described in the main text. Red lines denote the original estimates from the dataset, used to conduct the simulations.
(DOCX)

S2 Fig Histograms of 10,000 estimated values under a simulation experiment which generated data using from the mean of posterior estimates of the SIRD model described in the main text. Red lines denote the original estimates from the dataset, used to conduct the simulations.
(DOCX)
