# Supplementary material for: Hidden variable models reveal the effects of infection from changes in host survival
Source: PLoS Comput Biol. 2023 Feb 22;19(2):e1010910. doi: 10.1371/journal.pcbi.1010910 (PMC9987815; doi:10.1371/journal.pcbi.1010910)
Supplement: S1 Table — (DOCX) [file pcbi.1010910.s001.docx]

S1 Table: Parameter definitions and estimates for the fly SID models.

| **Variable** | **Prior distribution** | **Description** | **Estimated mode (SD**) |
| --- | --- | --- | --- |
| $\beta_{transfer}$ | Norm(0, 1) | The effects of days since the last vial transfer on fly survival | 0.28 (0.02) per day |
| $\beta_{age}$ | Norm(0,1) | The effect of time on fly survival | 0.11 (0.02) per day |
| $\alpha_{S}$ | Norm(0, 0.01) | Time-independent probability of survival of symptomatic flies | -7.10 (0.53) |
| $\alpha_{C}$ | Norm(0, 0.01) | Time-independent probability of survival of DCV symptomatic flies. | -3.09 (6.14) |
| $\alpha_{X}$ | Norm(0, 0.01) | Time-independent probability of survival of DXV symptomatic flies. | -4.27 (1.73) |
| $\pi_{C}$ | Uniform(0, 1) | Daily probability that an individual becomes infected with DCV. | 0.01 (0.01) |
| $\pi_{X}$ | Uniform(0, 1) | Daily probability that an individual becomes infected with DXV. | 0.02 (0.01) |
| $\tau_{S}$ | Gamma(0.01, 0.01) | Precision of random effect for vials in mock treatment | 0.0031 (0.39) |
| $\tau_{C}$ | Gamma(0.01, 0.01) | Precision of random effect for vials in DCV treatment | 0.0038 (5.56) |
| $\tau_{X}$ | Gamma(0.01, 0.01) | Precision of random effect for vials in DXV treatment | 0.002 (0.35) |
