# Supplementary material for: Hidden variable models reveal the effects of infection from changes in host survival
Source: PLoS Comput Biol. 2023 Feb 22;19(2):e1010910. doi: 10.1371/journal.pcbi.1010910 (PMC9987815; doi:10.1371/journal.pcbi.1010910)
Supplement: S2 Table — (DOCX) [file pcbi.1010910.s002.docx]

S2 Table: Parameter definitions and estimates for the seal SIRD models.

| **Variable** | **Prior distribution** | **Description** | **Estimated mode (SD**) |
| --- | --- | --- | --- |
| $\mu_{S}$ | Norm(0, 0.01) | Mean of the weekly number of strandings of uninfected individuals | $2.2\cdot{10}^{-4} (0.4\cdot{10}^{-4})$ strandings/week |
| $\omega$ | Unif(0, 13) | Time until stranding of an infected individual | $3.11(2.39)$ weeks |
| $\gamma$ | Unif(0, 13) | Time to recovery of infected seals | $2.58 (1.65)$ weeks |
| $\beta$ | Unif(0, 26) | Transmission period of an infected seal | $0.96 (0.14)$ weeks |
| $I_{0}$ | Unif(0, 500) | Initial number of infected individuals | $16.75 (20.05)$ individuals |
| $\tau$ | Gamma(0.01, 0.01) | Size parameter in negative binomial distribution | 2.73 (1.09) |
