# Supplementary figures and images for: Hidden variable models reveal the effects of infection from changes in host survival
Source: PLoS Comput Biol. 2023 Feb 22;19(2):e1010910. doi: 10.1371/journal.pcbi.1010910 (PMC9987815; doi:10.1371/journal.pcbi.1010910)

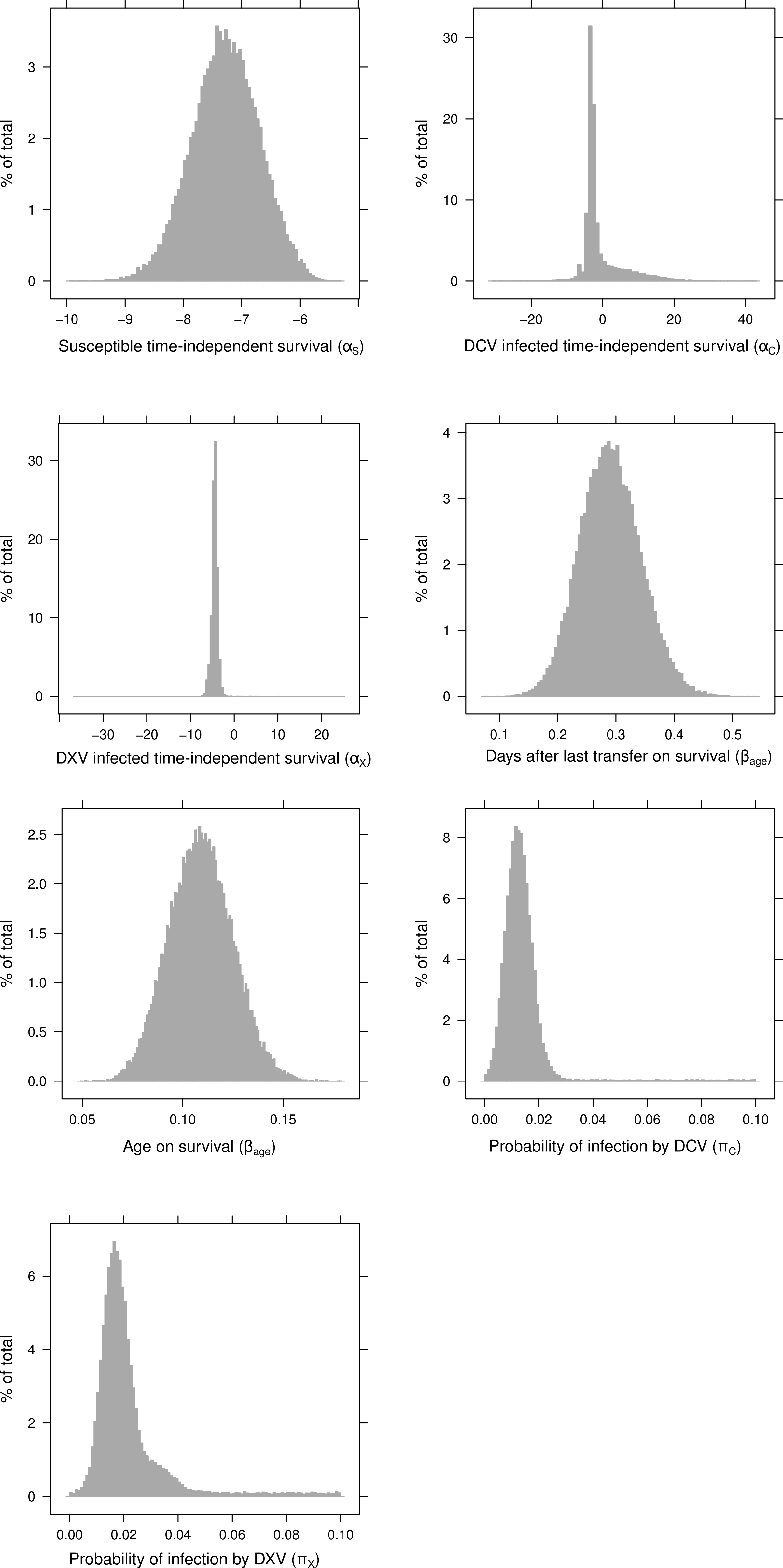


S Fig – Posterior distributions for parameters in the fly experiment survival model.

Supplement: S1 Fig — Red lines denote the original estimates from the dataset, used to conduct the simulations. (DOCX) [file pcbi.1010910.s003.docx]

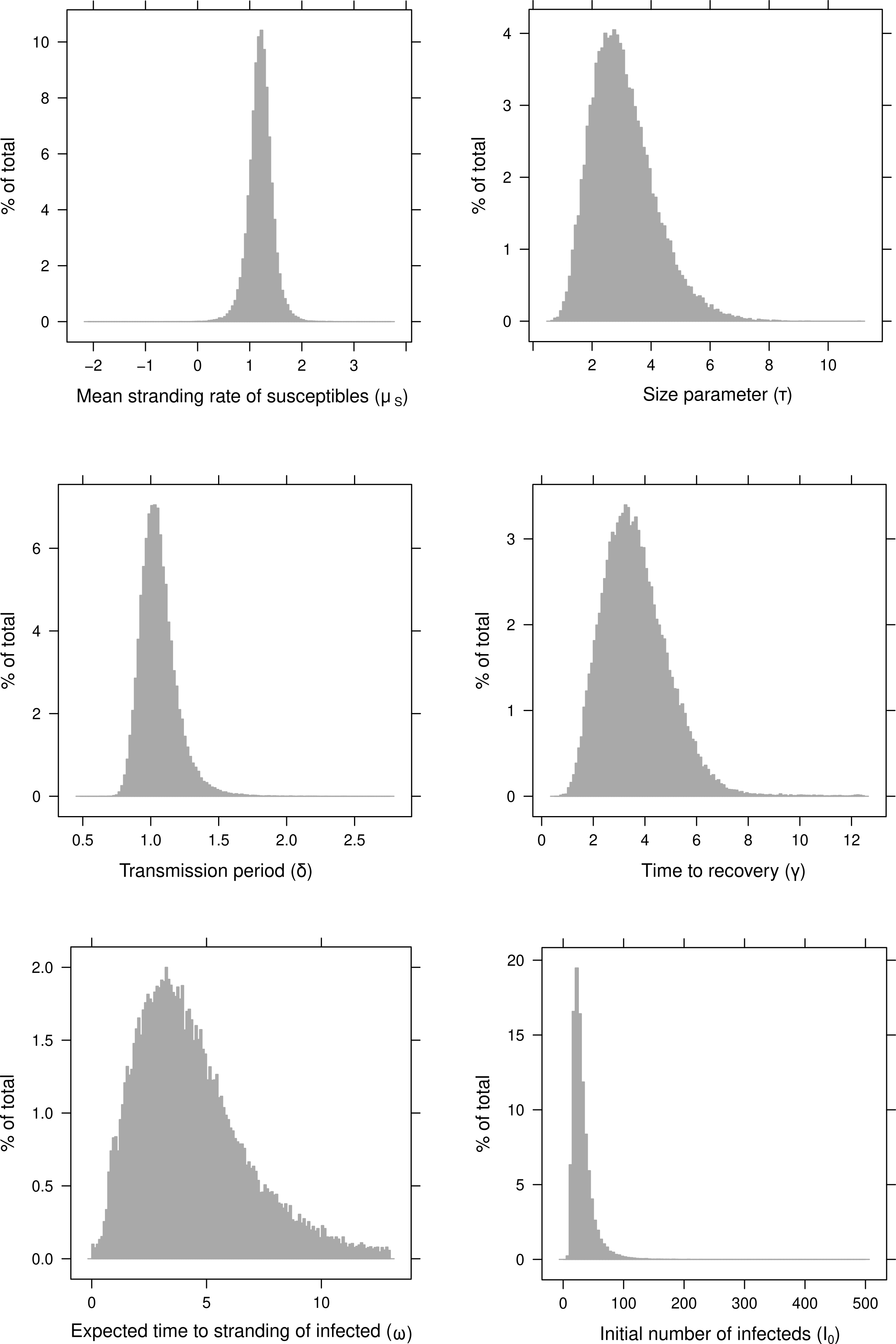


S Fig - Posterior distributions for parameters in the phocine distemper model.

Supplement: S2 Fig — Red lines denote the original estimates from the dataset, used to conduct the simulations. (DOCX) [file pcbi.1010910.s004.docx]
